# A Novel *HSF4* Gene Mutation Causes Autosomal-Dominant Cataracts in a Chinese Family

**DOI:** 10.1534/g3.113.009860

**Published:** 2014-03-17

**Authors:** Huibin Lv, Chen Huang, Jing Zhang, Ziyuan Liu, Zhike Zhang, Haining Xu, Yuchen You, Jinping Hu, Xuemin Li, Wei Wang

**Affiliations:** * Department of Ophthalmology, Beijing University Third Hospital, Beijing, 100191, China; † Medical Research Center, Beijing University Third Hospital, Beijing, 100191, China; ‡ Department of Ophthalmology, WeiHaiWei People's Hospital, 264200, Shandong China

**Keywords:** congenital cataract family, *HSF4*

## Abstract

Congenital cataracts are a significant cause of visual impairment or blindness in children. One-third of cases estimated to have a genetic cause. We carried out gene analysis and bioinformatics analysis to map the locus and to identify the underlying genetic defect in a 12-member, four-generation Chinese family affected with bilateral congenital cataracts. We screened individuals of the family and discovered a distinct missense mutation in *HSF4* (a gene at this locus that encodes teat-shock transcription factor 4). Bioinformatics analysis was used to determine possible changes in the protein structure that could affect the phenotype. Sequencing of the candidate genes showed a heterozygous c.69 G→T change in the heat shock transcription factor 4 (*HSF4*) gene, which resulted in the substitution of a lysine with an asparagine (p. K23N). This mutation cosegregated with all affected individuals and was not observed in unaffected family members. Bioinformatics analysis indicated that the p. K23N mutation was predicted to be disease causing. This is the first report of the novel missense mutation, c.69 G→T (p. K23N), in exon 3 of the *HSF4* locus on 16q21-q22 associated with bilateral congenital cataracts in a Chinese family. This novel mutation could enable propergenetic diagnostics and counseling in affected families and could lead to a better understanding of the structure and function of HSF4 in health and disease.

Congenital cataracts are a significant cause of visual impairment or blindness in children. The prevalence of congenital cataracts is 1 to 6 per 10,000 live births, depending on the method of ascertainment (Holmes *et al.* 2003). Globally, congenital cataracts account for nearly one-tenth of childhood blindness (Reddy *et al.* 2004a). Statistical analyses have revealed that congenital cataracts account for more than 1 million blind children in Asia. Approximately 50% of all congenital cataract cases may have a genetic cause (Francis *et al.* 2000; Rahi and Dezateux 2000; Santana and Waiswo 2011). Genetically, the majority of isolated congenital cataracts exhibit as autosomal dominant, although autosomal-recessive and X-linked inherited forms have also been reported (Vanita and Singh 1999).

During the past few years, remarkable progress has been made toward our understanding of the cataractogenesis process. More and more genes related to congenital cataracts have been mapped. So far, more than 40 loci have been mapped in congenital cataracts (Hejtmancik 2008; Zhao *et al.* 2011; Ouyang *et al.* 2012), and more than 26 genes have been characterized. Meanwhile, the number of associated genes is constantly increasing (Shiels and Hejtmancik 2007). Approximately one-half of the mutations are in the crystallin genes, and one-quarter are in the connexin genes. The remaining mutations are found in genes that encode heat shock transcription factor4 (*HSF4*), aquaporin-0 (*AQP0*, *MIP*), paired-like homeodomain 3 (*PITX3*), chromatin-modifying protein (*CHMP4B*), lens intrinsic membrane protein 2 (*LIM2*), beaded filament structural protein-2 (*BFSP2*), and other proteins (Reddy *et al.* 2004a; Devi *et al.* 2008).

According to their morphology, the cataracts can be classified into several subtypes: whole lens, nuclear, lamellar, cortical, polar, sutural, pulverulent, cerulean, coralliform, and other minor subtypes (Reddy *et al.* 2004b). It is known that different mutations in different genes could result in similar cataract patterns, while the highly variable cataract morphologies within some families suggest that the same mutation in a single gene can lead to different phenotypes (Heon *et al.* 1999; Gill *et al.* 2000).

In this study, a four-generation family affected with congenital polymorphic cataracts was investigated in an attempt to identify the genetic defect associated with their cataract phenotype. We applied a functional candidate approach to test the known characterized genes in this family. A novel missense mutation c.69 G→T (p. K23N) in *HSF4* was detected.

## Methods

### Clinical examination and isolation of genomic DNA

**Figure 1:**
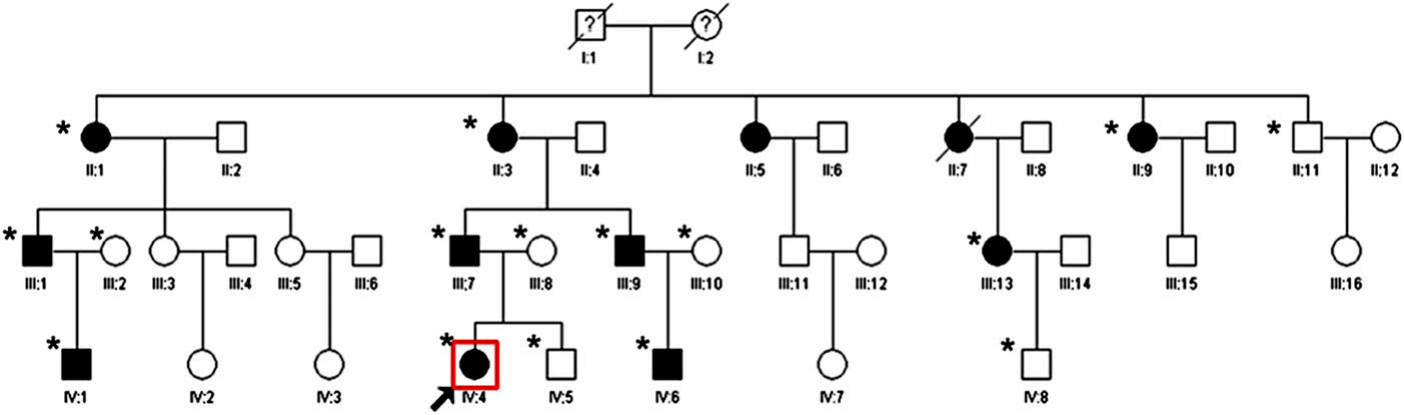
Pedigree of a cataract family. Pedigree of a four-generation family with congenital cataract. The proband is marked with an arrow. Squares and circles indicate males and females, respectively. Black and white symbols represent affected and unaffected individuals, respectively. The asterisks indicate family members who attend this study.

The proband, a 7-yr-old child, was diagnosed with bilateral cataracts at the Beijing University Third Hospital. A family history revealed 12 members in four generations. Sixteen people in this big family (II:1, II:3, II:9, II:11, III:1, III:2, III:7-10, III:13, IV:1, IV:4-6, IV:8) were willing to take part in the study (10 affected and 6 unaffected, Figure 1). The ethics committee of Beijing University approved the research, and all participants from the family provided their informed consent. The study protocol followed the principles of the Declaration of Helsinki. All participants were determined by a medical history or ophthalmologic examination, which included visual acuity, slit-lamp examination, ultrasonography, intraocular pressure measurement, and fundus examination with dilated pupils. Meanwhile, 100 unrelated ethnically matched control patients with no family history of congenital cataracts were recruited. Five milliliters of venous blood was collected from participating family members and controls in BD Vacutainers (BD, San Jose, CA) containing EDTA. Genomic DNA was extracted using QIAamp DNA Blood Mini Kits (QIAGEN Science, Germantown, MD).

### Mutation analysis

Twenty-one genes, including BFSP2, CRYAA, CRYAB, CRYBA1, CRYBB1, CRYBB2, CRYGC, CRYGD, CRYGS, EPHA2, GJA3, GJA8, HSF4, LIM2, MAF, MIP, PITX3, VIM, AGK, CHMP4B, and GALK1, were considered as candidate genes for hereditary cataracts (Reddy *et al.* 2004a; Hejtmancik 2008; Wang *et al.* 2011). The coding regions of the candidate genes were amplified by polymerase chain reaction (PCR) with previously published primer sequences (Litt *et al.* 1997; Hansen *et al.* 2006; Vanita *et al.* 2006; Lu *et al.* 2007; Shiels *et al.* 2007; Zhang *et al.* 2007; Schmidt *et al.* 2008; Bremond-Gignac *et al.* 2010) (supporting information, Table S1) and screened for mutations on both strands using bidirectional sequencing. Direct sequencing of the 100 ethnically matched controls was used to screen any identified mutations in the genes to confirm the mutations. PCR products were pooled, mixed with loading dye containing internal size standards, denatured at 95° for 5 min, and electrophoresed on 4% denaturing polyacrylamide gels on a DNA sequencer (ABI-Prism 377; ABI, Foster City, CA). The sequencing results were analyzed using Chromas 2.33 and compared with the reference sequences in the NCBI database.

### Bioinformatics analysis

The multiple-sequence alignment of the amino acid sequence in the mutated gene from several different species was analyzed by the CLC Free Workbench 6.0 software (CLC bio, Aarhus, Denmark). The three-dimensional (3D) structures of both wild-type and mutant proteins were predicted and analyzed by the online SWISS-MODEL tool (http://swissmodel.expasy.org/) (Guex and Peitsch 1997; Schwede *et al.* 2003; Arnold *et al.* 2006). The possible impact of an amino acid substitution on the structure and function of the protein was predicted by PolyPhen-2 (http://genetics.bwh.harvard.edu/pph2/) (Adzhubei *et al.* 2010) and Mutation Taster (http://www.mutationtaster.org) (Schwarz *et al.* 2010).

## Results

### Clinical findings

**Figure 2:**
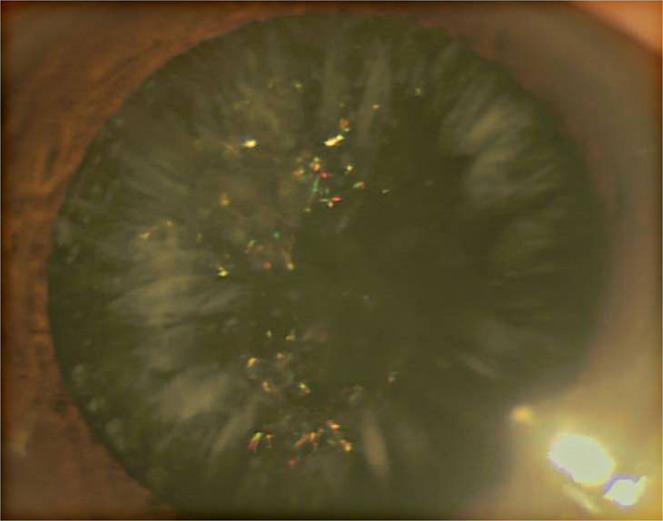
Slit-lamp photograph of the proband. The photograph of the proband (IV:4) shows rod-like opacities located in the lens cortex and colorful dots opacities in nucleus.

We have identified a four-generation Chinese family (16 members) in which 10 family members have been diagnosed with bilateral autosomal-dominant cortical cataracts (Figure 1). The proband (IV:4) was a 7-yr-old girl who was diagnosed with bilateral congenital cataracts. The rod-like opacities were primarily located in the lens cortex, and colorful dot opacities were seen in nucleus (Figure 2).

According to the medical records, except for the proband IV:4, eight affected individuals (II:1, II:9, III:1, III:7, III:9, III:13, IV:1, IV:6) had a cataract extraction performed between the ages of 7 and 25 years. The other affected patient had similar bilateral lens opacifications as well as some age-related lens nucleus opacities. The clinical evaluation of the affected individuals is provided in Table 1. Before surgery, the affected members had visual acuity ranging from 0.05 to 0.6. After surgery, all patients achieved a best-corrected visual acuity of 0.6−1.0. There were no other ocular or other related systemic abnormalities in this family.

**Table 1:** Clinical features of affected individuals

Affected Individual	Gender	Age	Age at Surgery	Phenotype
II1	Female	65	25	Aphakia eye, after cataract surgery
II3	Female	60	−	Rod-like cortical cataract with nuclear opacities
II9	Female	51	20	Aphakia eye, after cataract surgery
III1	Male	46	25	IOL, after cataract surgery
III7	Male	36	13	IOL, after cataract surgery
III9	Male	34	13	IOL, after cataract surgery
III13	Male	34	12	IOL, after cataract surgery
IV1	Male	17	11	IOL, after cataract surgery
IV4	Female	7	7	Rod-like cortical cataract with colorful dots opacities in nucleus
IV6	Male	11	8	IOL, after cataract surgery

Besides the proband IV:4, 8 affected individuals had a cataract extraction performed between the ages of 7 and 25 yr. IOL, intraocular lens.

### Mutation analysis

**Figure 3:**
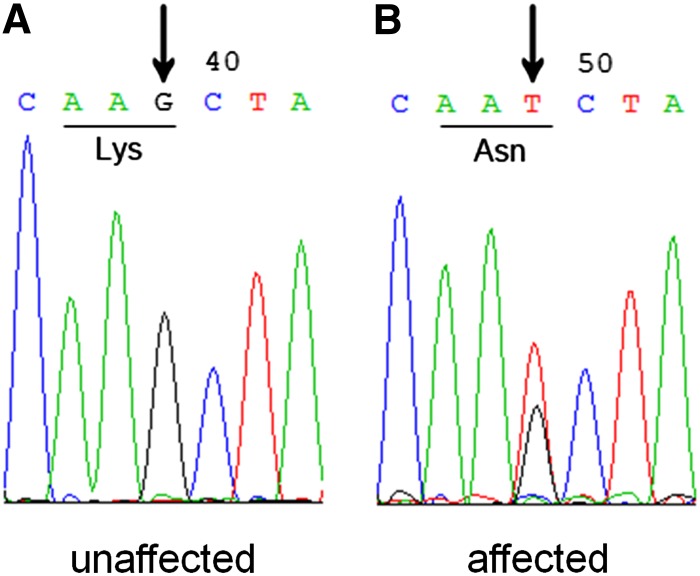
DNA sequence chromatograms of an affected and an unaffected individuals in the autosomal-dominant congenital cataract Chinese family (Forward strand; individual III:1 and III:2, respectively). A single transversion is observed at position 69(G→T) as a G/T double peak (indicated by a black arrow). (A) represents the unaffected gene sequence. (B) represents the affected gene sequence.

The 21 candidate genes were analyzed by sequence analysis of the coding regions. Bidirectional sequence analysis of the *HSF4* gene indicated a novel heterozygous c.69G→T variation in all 10 affected individuals of the family (Figure 3). This heterozygous mutation was not present in the unaffected family members or in 100 controls without congenital cataracts. This c.69G→T nucleotide alteration resulted in the substitution of a lysine with anasparagine (p. K23N). We did not find any other mutations in this family, except for a few nonpathogenic single-nucleotide polymorphisms (Table 2).

**Table 2:** All SNPS that have been found in all family members

Family Members	Cataract	*GJA3* p.L299M	*CRYBB2* p R61T	*CRYBB2* p.Q147R	*CRYBB2* p.T150M	*HSF4* p.K23N
II1	Yes	+	+	+	+	+
II3	Yes	+	+	+	+	+
II9	Yes	+	+	+	+	+
II11	No	+	−	−	−	−
III1	Yes	+	+	+	+	+
III2	No	+	−	−	−	−
III7	Yes	+	+	+	+	+
III8	No	+	−	−	−	−
III9	Yes	+	−	−	−	+
III10	No	+	−	−	−	−
III13	Yes	+	+	+	+	+
IV1	Yes	+	+	+	+	+
IV4	Yes	+	+	+	+	+
IV5	No	+	+	+	+	−
IV6	Yes	+	+	+	+	+
IV8	No	+	+	+	+	−

All of the single-nucleotide polymorphisms (SNPS) have been found in this family are shown. p.K23N is the only mutation that cosegregated with all affected individuals and was not observed in unaffected family members.

### Bioinformatics analysis

**Figure 4:**
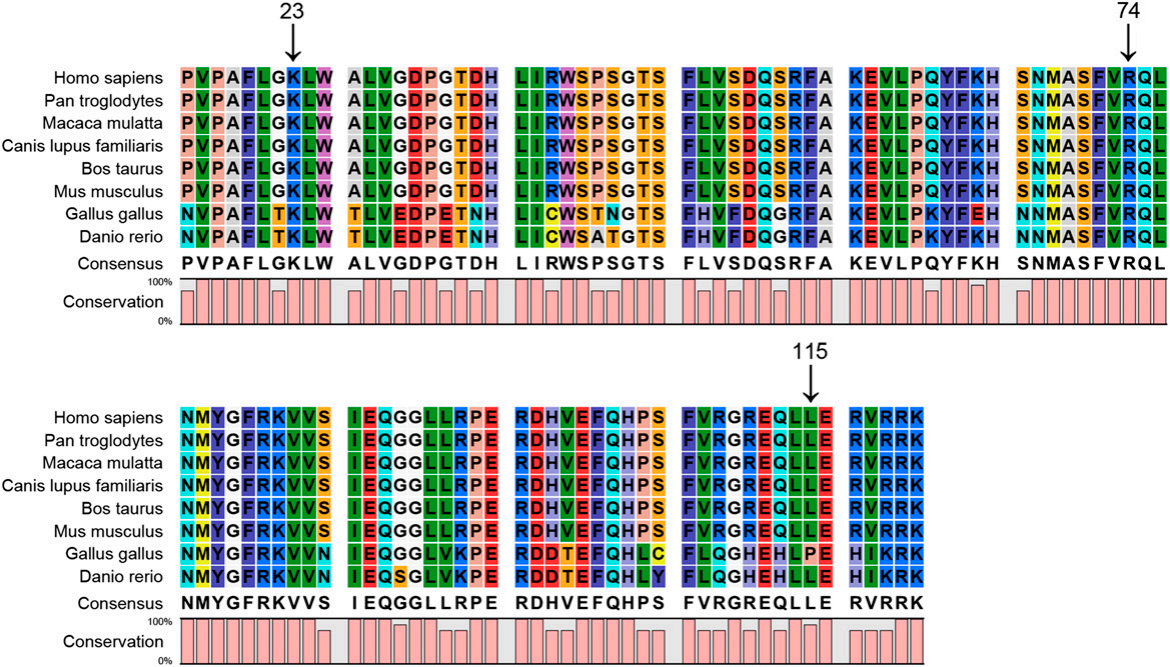
A multiple-sequence alignment in HSF4 (16−120) from different species. The alignment data indicate that the Phe at position 23 is highly conserved in different species (indicated by an arrow). Both amino acids at positions 74 and 115, known as causes of autosomal dominant cataracts, are also highly conserved in different species (indicated by an arrow).

**Figure 5:**
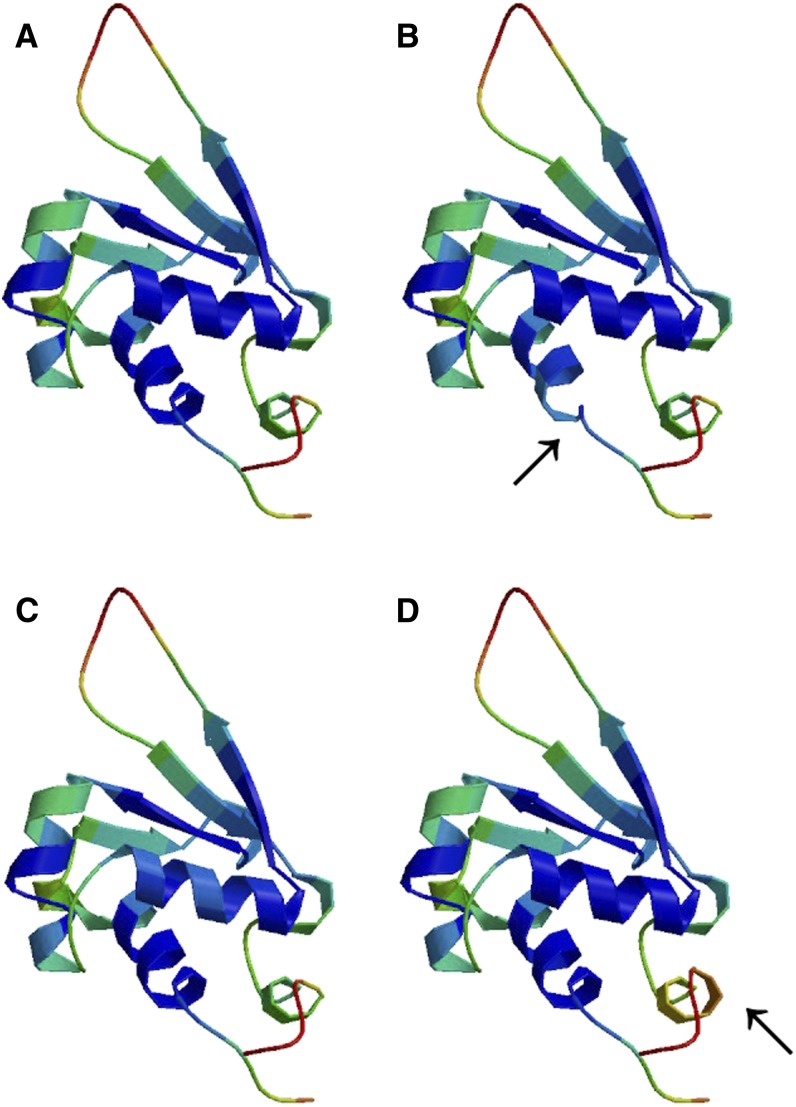
The three-dimensional structural models of the wild-type (A), the novel p.K23N mutant (B), the known p.R74H mutant (C), and p.L115P (D) HSF4 proteins. The modeled residue range is from amino acids 13 to 124 for all proteins. The novel p.K23N mutant (B) HSF4 protein and known p.L115P mutant (D) HSF4 protein represent observed different modeled structures with the wide-type one (indicated by an arrow).

The p. K23N (c.69 G->T) mutation in the HSF4 gene detected in our present study was located within the highly conserved HSF DNA binding region, which is shared across heat shock transcription factors (HSFs) and between species, as shown by multiple-sequence alignment (Figure 4). The modeled residue range extended from amino acids13 to 124. As shown in Figure 5, the predicted 3D structural model of the p.K23N mutated HSF4 protein was different from that of the wild-type protein. Additionally, the p.K23N mutation was predicted to be “probably damaging” by PolyPhen-2 analysis with a score of 1.000 and was predicted to be “disease causing” by Mutation Taster analysis.

## Discussion

In the present study in an autosomal-dominant congenital cataract family with 10 affected members in four generations, 21 known candidate genes were sequenced by direct sequencing using PCR, but 20 of them were excluded as pathogenic. The coding regions of these candidate genes were sequenced bidirectionally. We observed anovelc.69 G→T variation in the *HSF4* gene in the individuals affected with bilateral congenital cataracts. The heterozygous mutation resulted in the substitution of a lysine with anasparagine (p. K23N). This mutation likely caused the cataracts since it segregated with the phenotype and was not detected in either the unaffected family members or the 100 ethnically matched controls.

HSF4 belongs to the family of heat shock transcription factors (HSFs) that regulate the expression of heat shock proteins (HSPs) and mediate the inducible transcription response. HSF4 regulates the expression of HSPs in response to different cellular stresses, such as oxidants, heavy metals, elevated temperature, and bacterial or viral infections. HSPs play an important role in the maintenance of the supramolecular organization of the lens protein (Bachi *et al.* 2002), which is essential for lens transparency. In human beings, HSF4 is widely expressed in the body, especially in the heart, brain, skeletal muscle, lung, and pancreas (Nakai *et al.* 1997). The human *HSF4* gene has at least two alternatively spliced transcripts, *HSF4*a and *HSF4*b. Both these forms of HSF4 protein have the same DNA-binding domain, which is important for the function of HSF4.

Recently, the *HSF4* gene has been reported to be responsible for both autosomal dominant and autosomal recessive cataracts (Bu *et al.* 2002; Smaoui *et al.* 2004; Ke *et al.* 2006). Congenital cataracts can partly be distinguished by the location and severity of the mutations. So far, at least seven different mutations in the *HSF4* gene have been detected (Forshew *et al.* 2005; Ke *et al.* 2006; Mellersh *et al.* 2006; Talamas *et al.* 2006). Two of them can lead to autosomal-dominant cataracts, another three can cause autosomal-recessive cataracts, and the remaining two mutations were found in sporadic cases. So far, all known dominant mutations in *HSF4* (p.R74H, p.L115P) are located in the α-helical DNA binding region, which further highlights the importance of this domain (Bu *et al.* 2002; Ke *et al.* 2006). In this study, the missense mutation (p.K23N) also lies within this highly conserved functional domain. However, the recessive mutations lie outside this highly conserved functional domain. Previously, Smaoui *et al.* (2004) reported a splice mutation in *HSF4* associated with an autosomal recessive total cataract in a Tunisian family.

Bioinformatics analyses were performed to elucidate a correlation between structural disturbances and putative functional commitment, achieving a possible explanation for the pathogenic mechanism of the novel p.K23N missense mutation of *HSF4*. The p.K23N mutation is located within a highly conserved region across species, which suggests an important role in the function and/or structure of HSF4. To evaluate the 3D impact of the mutation, we created a homology model to compute and compare mutant and wild-type structures. The spatial structures of both wild-type and mutant proteins were modeled by the online SWISS-MODEL tool (http://swissmodel.expasy.org/), base on temple: 2lduA (99.9 A). The 3D structural models also indicated the different structure between the wild-type and p.K23N mutant proteins. Moreover, the p.K23N mutation was predicted to be “probably damaging” by PolyPhen-2 analysis with a score of 1.000 and was predicted to be “disease causing” by Mutation Taster analysis. Hence, the predicted change of the protein structure could disrupt lens biochemistry and physiology early in development. The possible mechanism of this mutation will require further investigation.

However, in this family, most patients, except the proband and II:2, had cataract extraction performed between the ages of 7 and 25 yr. The details of the phenotypes of the other individuals could not be acquired. It is not certain whether the lens opacities of all affected family members were similar. It is also unknown whether the opacity of the lenses worsened with age. Despite this, this novel mutation in the *HSF4* gene could provide some clues to the mechanism of developing congenital cataracts.

Moreover, Bagchi *et al.* (2002) revealed that certain sequence changes in *HSF4* resulted in abnormal expression of HSPs and thereby influenced the function or level of HSPs. The decrease of HSPs could be responsible for the loss of optimal protein organization and the eventual appearance of age-related cataracts (Shi *et al.* 2008). In addition, regarding the clinical phenotype caused by mutations in this transcription factor, the reason that mutations in *HSF4* that are expressed in other tissues, including the heart, muscle, lung, and brain, cause only nonsyndromic cataracts (Smaoui *et al.* 2004) is still unknown. More comprehensive studies will be needed to answer this question.

In summary, we have shown a novel missense mutation in *HSF4* that mapped to 16q21-22 and caused autosomal-dominant cataracts in a large Chinese family. Sequencing of the candidate genes showed a heterozygous c.69 G→T variation in the *HSF4* gene, which resulted in the substitution of a lysine with anasparagine (p. K23N). This novel mutation could enable proper genetic diagnostics and counseling in affected families and could lead to a better understanding of the structure and function of HSF4 in health and disease.

## 

## Supplementary Material

Supporting Information
