# Supplementary material for: A Novel HSF4 Gene Mutation Causes Autosomal-Dominant Cataracts in a Chinese Family
Source: G3 (Bethesda). 2014 Mar 17;4(5):823–8. doi: 10.1534/g3.113.009860 (PMC4025481; doi:10.1534/g3.113.009860)
Supplement: Supporting Information [file supp_4_5_823__index.html]

A Novel HSF4 Gene Mutation Causes Autosomal-Dominant Cataracts in a Chinese Family — Supporting Information 

# A Novel *HSF4* Gene Mutation Causes Autosomal-Dominant Cataracts in a Chinese Family

## Supporting Information for Lv *et al.*, 2014

**Files in this Data Supplement:**

- Table S1 - Polymerase chain reaction primers and product sizes. (PDF, 168 KB)
