# Supplementary material for: A Novel HSF4 Gene Mutation Causes Autosomal-Dominant Cataracts in a Chinese Family
Source: G3 (Bethesda). 2014 Mar 17;4(5):823–8. doi: 10.1534/g3.113.009860 (PMC4025481; doi:10.1534/g3.113.009860)
Supplement: Supporting Information [file supp_g3.113.009860_009860SI.pdf]

G3 · Genes | Genomes | Genetics

A novel *HSF4* gene mutation causes autosomal dominant cataracts in a Chinese family

Huibin Lv*[1], Chen Huang*[2], Jing Zhang[1], Ziyuan Liu[1], Zhike Zhang[1], Haining Xu[3], Yuchen You[1], Jinping Hu[1], Xuemin Li[1] & Wei Wang[1]
1 Department of Ophthalmology, Beijing University Third Hospital, Beijing, China
2 Medical Research Center, Beijing University Third Hospital, Beijing, China
3 Department of Ophthalmology, WeiHaiWei People`s Hospital, Shandong China

*Huibin Lv and Chen Huang contributed equally to this work.
Corresponding to: Professor Xuemin Li, Department of Ophthalmology, Beijing University Third Hospital, Hua Yuan Bei Lu, Beijing 100191, China. Phone: +8610-82266573; email: lxmlxm66@sina.com.cn

DOI: 10.1534/g3.113.009860

Table S1 Polymerase chain reaction primers and product sizes.

Name	Primer name	Primer sequence (5′-3′)	Product size (bp)
CRYAA	CRYAA-E1f	CCATTCTGCTGGTGGCATATA	436
	CRYAA-E1r	CCTCTGCAAGGGGATGAAGT	
	CRYAA-E2f	AGGTGACCGAAGCATCTCTG	494
	CRYAA-E2r	GAAGGGACCCTAACCTGGAT	
	CRYAA-E3f	CCCCCTTCTGCAGTCAGTG	402
	CRYAA-E3r	GGAAGCAAAGGAAGACAGACAC	
CRYAB	CRYAB-E1f	CATATATAAGGGGCTGGCTGTAG	369
	CRYAB-E1r	GGTTTAGGCAGGGTAGGAAAG	
	CRYAB-E2f	GAATTACCCGGACAGAAAGCA	322
	CRYAB-E2r	GAATGTAGCCAGCCTCCAAAG	
	CRYAB-E3f	GTCTCACCTAAGGGGAAATCAG	400
	CRYAB-E3r	GCCCTTAGCATTAATAAGCTTCA	
CRYBA1	CRYBA1-E1f	GGAACAAAGGCAGAGGGAGA	248
	CRYBA1-E1r	CACTCTTCTCCAGCCACCCTA	
	CRYBA1-E2f	AGTGAGCAGCAGAGCCAGAA	293
	CRYBA1-E2r	GGTCAGTCACTGCCTTATGGAC	
	CRYBA1-E3f	TGGTAACAGAAAGCACAGAGTCA	320
	CRYBA1-E3r	CCTAGGGGAACATCCTTCTTC	
	CRYBA1-E4f	TGGGATTGGCTTGATATTTTAC	364
	CRYBA1-E4r	CCTGCCACATTCAGCATAACT	
	CRYBA1-E56f	ATGAAGAATGATAGCCATAGCACT	827
	CRYBA1-E56r	GAGTGCTTAGCAAGGTCTCATG	
CRYBB1	CRYBB1-E1f	CCCTGAAGGCCAGAGTTTCT	331
	CRYBB1-E1r	CGGAGGAGTAAGAGGTGAAAGA	
	CRYBB1-E2f	TTATGTGACTCCTGCACTGCTG	296
	CRYBB1-E2r	GGAGAAATGGCAGCTACTGTTG	
	CRYBB1-E3f	CCCCTTTGGACTTTCCTACTGT	335
	CRYBB1-E3r	CCCTTGTCAGATCTCAGACTTACA	
	CRYBB1-E4f	CCCGCTAAGTTTCTTCTCTTTG	269
	CRYBB1-E4r	CCTCTGATTCTGCCTGTGCT	
	CRYBB1-E5f	GCAGGGATCAATGAAGGACAG	338

	CRYBB1-E5r	GGGAAATAATTGAACATGAAGAAG	
CRYBB2	CRYBB2-E1f	CCACAGAGTGAAAAAGCCAGTG	236
	CRYBB2-E1r	TACCCATGAAGAAAGATGCCA	
	CRYBB2-E2f	CATCCTTTGGGTTCTCTGAGC	278
	CRYBB2-E2r	ACTCCACAACTTGATCACCTCAC	
	CRYBB2-E3f	TTTGGGTGGGGCTATTACATC	309
	CRYBB2-E3r2	GATGGGCAGAGAGAGGGAGTA	
	CRYBB2-E4f	GACATGCTGATCCCAACTCTG	343
	CRYBB2-E4r	GATGTGTGCAAGATCACCAGC	
	CRYBB2-E5f2	CTCGTTCACCCTCCCATCA	304
	CRYBB2-E5r2	CAGACAAGTTGCAAGTCACACTTT	
CRYGC	CRYGC-E12f	TGCTATATAGACTGGCTGTGCAG	492
	CRYGC-E12r	CCCTCCCTGTAACCCACATT	
	CRYGC-E3f	GTTCTTTGGTTGGACAAATTCTG	411
	CRYGC-E3r	TTGCCAGCAATGCAGACTAAAT	
CRYGD	CRYGD-E12f	GTTCTTGCCAACGCAGCAG	531
	CRYGD-E12r	GCTTGAAACCATCCAGTGAGTG	
	CRYGD-E3f	AAGCTGGACTGCCTAACAATG	415
	CRYGD-E3r	TGCCAGGAACACACAGAAAAT	
CRYGS	CRYGS-E1f	GTCACTGTGCCTCTAACTCTCTGT	221
	CRYGS-E1r	CTCAATCCCAGTTCTCTAACCAC	
	CRYGS-E2f	TCTGTGAATTAAGCCACCCAG	388
	CRYGS-E2r	GAAGACTCAAGAAAATGTTCACCT	
	CRYGS-E3f	ACCTGCTGGTGATTTCCATAAT	418
	CRYGS-E3r	CATGCCAACTGTTTTATTGATGAT	
BFSP2	BFSP2-E1f	CCAAAAGCCACTGGACTCTGT	623
	BFSP2-E1r	CTACTCACAACCCTACCCGCT	
	BFSP2-E2f	TAAGTGATCTTGACGCTCCCA	190
	BFSP2-E2r	AGGAGTCTAGGGATGTGGAGG	
	BFSP2-E3f	TGATTCTTTTTCTTTTGGGCTAC	276
	BFSP2-E3r	ACAGGCAGACAGATGTGAACTTC	
	BFSP2-E4f	AGCTGGTTTTCTGCTGGCTA	312
	BFSP2-E4r	ACTAGTTTGCAGCTCAGCGTG	
	BFSP2-E5f	AAGGATGGGCTCAGATTTCTG	286
	BFSP2-E5r	GGAATCCCCTGGAAACTAAGA	

	BFSP2-E6f	TCTGCACACCTCCCTCTTCA	402
	BFSP2-E6r	GGAAGTCTGGGGTGATTTCTT	
	BFSP2-E7f	GAATGACCGATTTTCCTCACC	174
	BFSP2-E7r	GAATTAACCAGGGAATCCAGC	
GJA3	GJA3-E1Af	CGGTGTTCATGAGCATTTTC	743
	GJA3-E1Ar	CCTGCTTGAGCTTCTTCCAG	
	GJA3-E1Bf	ACGGTGGACTGCTTCATCTC	810
	GJA3-E1Br	GCACTTTGGTTTTGGTTTCTAA	
GJA8	GJA8-E1Af	ATATTGACTCAGGGTTGCATTG	783
	GJA8-E1Ar	TCTTCAAGGCAGACCGGAT	
	GJA8-E1Bf	TGTGGCCTCTGTGTCCCTAT	772
	GJA8-E1Br	TGGGAGGACAGGAGACAGAA	
MIP	MIP-E1f	CCTCTATAAAGGGGACTGTCCA	506
	MIP-E1r	GTCAGGGAGTCAGGGCAATA	
	MIP-E23f	GAGGAGGTAACACTGTGGCAG	805
	MIP-E23r	GAACCTGCAGTCCACAACCA	
	MIP-E4f	AAGAGCAGCGTTGCTACTCTG	299
	MIP-E4r	CCCTCCACGTAAACTCAGAAG	
VIM	VIM-E1f	TCTTCTCCGGGAGCCAGTC	662
	VIM-E1r	TCCCGAGGCCCAGCTACTT	
	VIM-E2f	GGTTTCCTCGTTCCCCTTTG	166
	VIM-E2r	GAATTGCTCGTGGGTTGTGT	
	VIM-E34f	AAGCCATACACTTTTACATCCTCC	455
	VIM-E34r	GGGTTTTGAGAACACAATGCTT	
	VIM-E56f	AGTCAAAAGACTTGAATGTGAGCA	804
	VIM-E56r	CAGGGCCTAACAAATGGTCAC	
	VIM-E78f	TTGCCAACAATTTTACTGTTTCTC	666
	VIM-E78r	TAGCCAGCTGTATCTGAGATTCAG	
	VIM-E9f	GGATAATTTAGTCTTTGGCATGTG	185
	VIM-E9r	GCACTTGAAAGCTGTTTCTTTAAG	
HSF4	HSF4-E1f	TTCCGCGGCTTTGACGAG	247
	HSF4-E1r	GTTCACTGACGTGGAGGGACC	
	HSF4-E234f	CGCTCACCCTCCTGGTCTC	669
	HSF4-E234r	CCGGCCAGTTATGGTCTCATC	
	HSF4-E56f	ACTGGGCGAACTCTCAGATG	404

	HSF4-E56r	ACTTCTGGGGGGTCTTGGA	
	HSF4-E7f	AGGGAGGGAAGTGCAGGC	
	HSF4-E7r	CCATAAGCCCAGCCATGC	
	HSF4-E89f	GGTTCTGGCTCTCCCTGTG	598
	HSF4-E89r	GAAGCTTTGTGGGCTGGTAA	
	HSF4-E1012f	GGGTTCCTTGGGAACTTAATG	639
	HSF4-E1012r	CAAGGTAGCTCAGCCCAATC	
	HSF4-E13f	GGCTCTCCTTCCCTGAAGAAAG	285
	HSF4-E13r	CAGGAAGCCAAGAAGGATGTG	
MAF	MAF-E1Af	CCTCTCCTGCAGCCCATCTG	455
	MAF-E1Ar	CGCGCGTAGCCATCGAAG	
	MAF-E1Bf	CGGTCGAGGCGCTCATCA	450
	MAF-E1Br	GGTTCAGCTCGCGCACAGAC	
	MAF-E1Cf	TGCACTTCGACGACCGCT	406
	MAF-E1Cr	CCCGCGGAGCACTTATCA	
	MAF-E2f	GCTGCGTTTGATCTTTGTCTAA	224
	MAF-E2r	GGTAGGTGGTTCTCCATGACTG	
PITX3	PITX3-E1f	ACCCCCATTACCCTGGTCTGT	216
	PITX3-E1r	GGGATGAAGCTGTTATGTCCTGC	
	PITX3-E2f	CTGGCTTGGGCGCTCTGT	332
	PITX3-E2r	AGGTGGGGTGGAACCGCT	
	PITX3-E3f	CCCTTCAGCCGCTGGGAC	702
	PITX3-E3r	GCGGAGGCTGTGAATCGTTG	
LIM2	LIM2-E1f	TCAGCTCCCAATCCCATCTC	398
	LIM2-E1r	CTGCAGCTAGAAAACAACCCTG	
	LIM2-E2f	GCAGCATCTCTCACTTAGAGCA	268
	LIM2-E2r	CAGATTGGGGTTTGAGATGAG	
	LIM2-E34f	CAAAATCACACCCAGCCTTAG	547
	LIM2-E34r	CTCTTCAGTGGCCTCACTTTAAC	
EPHA2	EPHA2-E1f	GGCAGGAGGGGCAGAAGTTG	290
	EPHA2-E1r	AGTTGCGCGCGTCAAGGAG	
	EPHA2-E2f	CCATGGTCTGACTCCTGAAGA	203
	EPHA2-E2r	GCCTCAGTTTCTCCATCTCTACA	
	EPHA2-E3f	AGGCACCTGCCCACACTAAC	764
	EPHA2-E3r	ACCAGAACCTGGGAATGCAG	

	EPHA2-E45f	GGGTGGAAGCAGATTGAACTGA	695
	EPHA2-E45r	TCCTCCTTAAGCCCCACCTG	
	EPHA2-E6f	CAAACCCTGCCTGCTCGTAG	267
	EPHA2-E6r	ATGGCTGGGTGGTTTGGTG	
	EPHA2-E7f	TGCCCTCACTGCCTGCTC	232
	EPHA2-E7r	CCCTGGCCTGGTCCATG	
	EPHA2-E8f	CGGGTGTTGCTGTAATTTGTG	221
	EPHA2-E8r	AGGTTCCTGCCCCATTTTC	
	EPHA2-E9f	CCGGAGCAGACCTCACTGAC	239
	EPHA2-E9r	CAGAGCTGCCCACGGAAG	
	EPHA2-E1011f	CGTGACCTTCTCCTCTGACTC	569
	EPHA2-E1011r	AGACAGAGCCCCTGCTAAGTG	
	EPHA2-E1213f	TGGGCCCACTTACCTCTCAC	487
	EPHA2-E1213r	GGTGTGCAGGTGAGAGGACAC	
	EPHA2-E14f	CTTGGCTGCAATGGTCCTG	289
	EPHA2-E14r	CCCCTGCAGTTTGAGATGAGT	
	EPHA2-E15f	TTCAGTGGCTTTCTGCAACAG	287
	EPHA2-E15r	CAGAAGGAAAATTGAGGTCATCA	
	EPHA2-E16f	AGGTTAGGGAGCAGCAGGTG	329
	EPHA2-E16r	ATTGAGGGGCAGGGAAGAG	
	EPHA2-E17f	CTGCTCCAGCCCCTAACTCT	212
	EPHA2-E17r	AGGGAGGCCACTCTGTTTCTT	
AGK	AGK-E2-F	GTAAGGAGGAAGAGGAGTGA	422
	AGK-E2-R	GTTCCAGCTCTGGTTACAAG	
	AGK-E3-F	TTACAGGCGTGAGCCACCAT	322
	AGK-E3-R	GCGTCAGAACTGCTCTTCAC	
	AGK-E4-F	CTGACAGACTATGGCCTTAC	725
	AGK-E4-R	GACAGAGATGGCCTAAGGAT	
	AGK-E5-F	CTAGCTCCTCCACCTGTGTC	660
	AGK-E5-R	GCCTCCATCCAATCCTATTC	
	AGK-E6-F	TCATGTCTATCAGTTATGTA	304
	AGK-E6-R	CAACATACCATTGCTCTAGG	
	AGK-E7-F	GTATATGTACTTCATAGTGG	563
	AGK-E7-R	TGTGTTAATATACGTGTTCT	
	AGK-E8-F	AGCACCTTCACCTGTCATTG	640

	AGK-E8-R	CTGATCTAGGTCCTCCTCTT	
	AGK-E9-F	GGCACAATGTAGAGACTCAG	474
	AGK-E9-R	GGTAGGAATGGAAGAGCAAC	
	AGK-E10-F	TCTAGCTGACCTGACTACTG	795
	AGK-E10-R	GACTGCAACAGCTCCGTAAG	
	AGK-E11-F	CTAAGCCATAACCTCCTAGC	308
	AGK-E11-R	CATGCCACATTCATCTGTAG	
	AGK-E12-F	AGCATTCCATCCAGTGAGAG	400
	AGK-E12-R	GAGGCTTCTCCTGCTCTAGT	
	AGK-E13-F	TGAAGGCAGAGTCAGCAGAA	366
	AGK-E13-R	AAGGCACACCACATCCTCTT	
	AGK-E14-F	AGTGACAGAACGTCCAGAAC	539
	AGK-E14-R	AGTGATGGCCAGCATAGGAG	
	AGK-E15-F	ATGGCTTAATAGACCATCTG	321
	AGK-E15-R	TGTGCCTAAGTCTAGGTCAG	
	AGK-E16-F	TTCCAGAACCAGCACTGT	524
	AGK-E16-R	GCGCTAATGGAAGCCGATTG	
CHMP4B	CHMP4B-E1-F	GCCTGAAGCCGGAAGACTAT	590
	CHMP4B-E1-R	CGAGGCGAGTCTGATGAA	
	CHMP4B-E2-F	TTCTTCCTCAGGTGGCTGTC	577
	CHMP4B-E2-R	ACTCAGGTGCTCGAATAGGT	
	CHMP4B-E3-F	CCTTGATAGTGCCACCTGTA	630
	CHMP4B-E3-R	TGCAGGCTAGCTACTTGTTC	
	CHMP4B-E4-F	GCAGGACACCAGAGGTCTAC	515
	CHMP4B-E4-R	CCAGACAGGATCCATTGTAG	
	CHMP4B-E5-F	TAGGCACCAAGGAGCAGTCT	324
	CHMP4B-E5-R	GAACCTGCCTGCACCACATC	
GALK1	GALK1-E1-F	GAACCGGCTGAGGTCTGG	459
	GALK1-E1-R	CGGTGCACGGAAGAGTCC	
	GALK1-E2-F	GTGCACAGGTACAGGATGTC	728
	GALK1-E2-R	GGCTCCTCCAATCAGGTCAC	
	GALK1-E3-4-F	AGCAGGTTGGTGGCTTCTGA	773
	GALK1-E3-4-R	GCGGACATTAGAGTTGGTGA	
	GALK1-E5-F	ACGCGCTGCTCATTGACT	626
	GALK1-E5-R	ATGTCAGCAGTGGCTATCTC	

GALK1-E6-7-8-F	CTTCACCGTCCAGCCAGG	769
GALK1-E6-7-8-R	TACCACATTGGAGGCACAAG	
